# The association between china’s primary health care reform and inequalities in primary care utilisation and maternal mortality: a quasi-experimental longitudinal study from 2010 to 2019

**DOI:** 10.1186/s12939-025-02541-z

**Published:** 2025-06-13

**Authors:** Chang Cai, Christopher Millett, Jin Xu, Yanshang Wang, Thomas Hone

**Affiliations:** 1 Department of Primary Care and Public Health, School of Public Health, Imperial College London, London, UK; 2 Public Health Research Centre and Comprehensive Health Research Centre, NOVA National School of Public Health, NOVA University, Lisbon, Portugal; 3 China Center for Health Development Studies, Peking University, Beijing, China; 4 School of Public Health, Peking University, 38 Xue Yuan Road, Haidian District, Beijing, 100191 China

**Keywords:** Primary health care, China, Longitudinal studies, Maternal health inequalities

## Abstract

**Background:**

China’s maternal health has substantial inequalities across regions, a similar challenge faced by many low- and middle-income countries. The Chinese government launched a comprehensive health reform since 2015 to deliver more affordable and equitable primary health care (PHC), with pregnant women being a priority group of beneficiaries. However, little is known about the impacts of this PHC reform on primary care utilisation among pregnant women or maternal health inequalities. This study aims to examine whether and how China’s PHC reform affected primary care utilisation among pregnant women and maternity deaths differently across regions.

**Methods:**

The study employed provincial-level panel data from the China Health Statistic Yearbook and China Statistic Yearbook (2010–2019). Reform implementation by province was identified using web-scrapping of 31 provincial government websites. Firstly, difference-in-differences method examined the reform impacts on visits to PHC facilities, the utilisation of family physician services and prenatal services, and the maternal mortality ratio (MMR). Secondly, fixed-effects panel regression models estimated the association between family physician service use, prenatal care and the MMR. Analyses were stratified by province human development index (HDI) to assess inequalities.

**Results:**

The introduction of China’s PHC reform in a province was associated with increased utilisation of family physician services (59.7 per 10,000 people per year, 95% CI 32.8–86.5) and prenatal services (3.2% points per year, 95% CI 1.8–4.6) and reduced maternal death by 9.6 per 100,000 live births per year (95% CI 0.3–19.0) in low-HDI provinces. No reform impact was found in high-HDI provinces. In panel regression models for low-HDI provinces, with a 1.0% point increase in prenatal care utilisation and one increase in family physician visit per 100 people, maternal deaths would decrease by 1.4 (95% CI 0.2–2.5) and 2.4 (95% CI 1.4–3.5) per 100,000 live births per year, respectively. This association was not found in high-HDI provinces.

**Conclusion:**

China’s PHC reforms and primary care utilisation were associated with reduction in MMR in less developed regions, suggesting contributions to lower inequalities in maternal health between regions. Community-level family physician services are likely effective for improving maternal health in high burden areas, but further system and quality improvements are needed in areas where maternal mortality is lower.

**Supplementary Information:**

The online version contains supplementary material available at 10.1186/s12939-025-02541-z.

## Introduction

Maternal health inequalities are prevalent in low- and middle-income countries (LMICs) [1–4], including China [5–7]. Women from poorer households, resource-restrained areas, and with lower educational attainment are disproportionally affected by inadequate access and quality to maternal care [2, 4, 8, 9] and consequently, high maternal mortality [1–4, 10]. However, many of the primary causes of maternal deaths in LMICs, such as hypertensive disorders and haemorrhage [11, 12], are often preventable with frequent maternal examinations and adequate treatments [13, 14]. By addressing the burden of maternal deaths in the vulnerable groups, LMICs can make significant progress toward reducing maternal health inequalities and the achievement of the Sustainable Development Goal of “reducing global maternal mortality ratio (MMR) to less than 70 maternal deaths per 100,000 live births by 2030” [13]. 

**Fig. 1:**
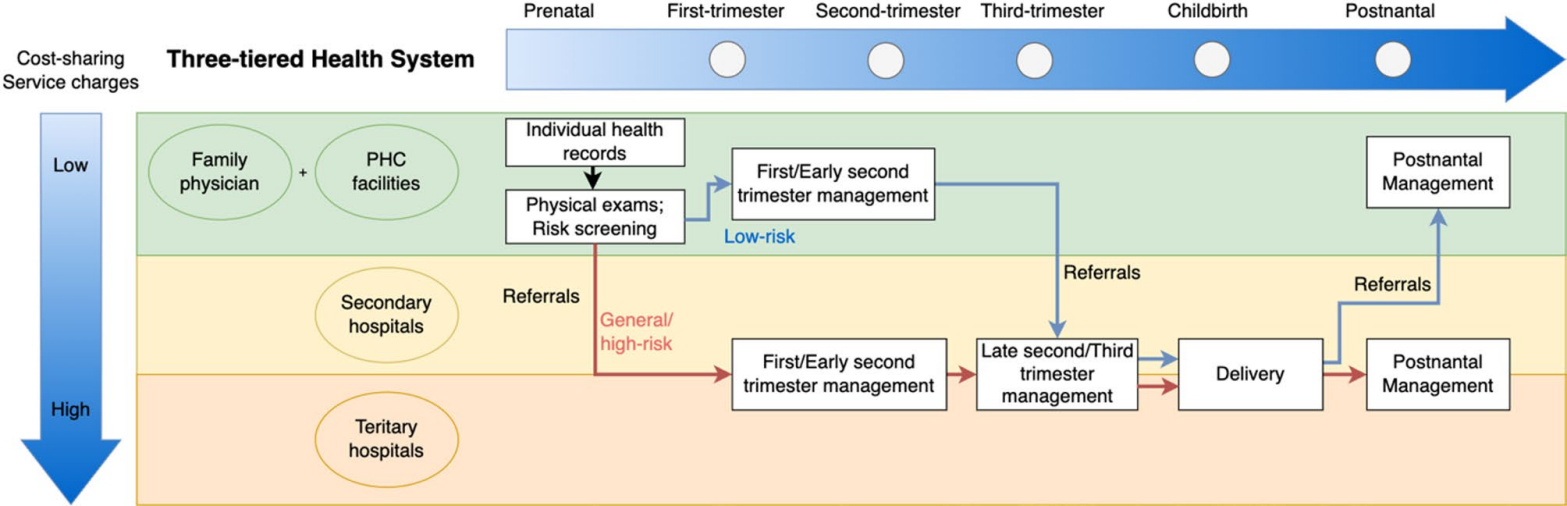
A diagram of the continuum of maternal care during prenatal, childbirth and postnatal periods in China’s three-tiered health system. Source: this diagram is made based on the Guidelines on the National Essential Public Health Services (2017 version) [33] and the Notice on Standards for Pregnancy Risk Assessment and Maternal Management (2017 version) [34]

China launched a nationwide health system reform since 2015 to deliver more affordable and equitable primary health care (PHC) with a priority on pregnant women [15]. The reform emphasized the crucial role of PHC in achieving equitable maternal health through three main components: First, a hierarchical health system with partial gatekeeping was established [16]. PHC facilities served as first contact for pregnant women for initial free-of-charge examinations, pregnancy risk assessments and booking for following care and referrals. For low-risk pregnancies, prenatal care was often provided by PHC providers (PHC facilities and family physicians) until 26 weeks before referral to higher-level hospitals and further screening tests were needed (Fig. 1). Second, family physicians were introduced to provide similar care to pregnant women as provided by PHC facilities, but at the community level [17]. Family physicians also provided home visits which allow pregnant women access essential maternal care without travelling to medical facilities [18]. Pregnant women were incentivised to first contact with PHC providers and enroll with family physicians by a higher reimbursement rate compared to hospitals, but these policies were not compulsory. Third, PHC facilities and hospitals were integrated into a two-way referral system with a shared standardised health information system [19]. All relevant personal information and health data were recorded throughout the pregnancy and the 42-day postnatal periods, allowing timely referrals and continuous maternal care.

The reform was implemented in a staggered manner. Six provinces piloted the reform in 2014, followed by another three provinces before the national roll-out in September 2015. Later, the other 22 provinces implemented the reform, with the last one joining the reform in 2017. This staggered implementation facilitates a natural quasi-experimental study to assess the reform impacts on primary care utilisation and maternal mortality. Whilst the selection criteria of pilot provinces is unknown, a previous study shows the staggered implementation is not associated with the demographical, socioeconomic or health system characteristics of the regions [20]. 

Despite the significance of China’s PHC reform, there is little understanding of how the reforms affected maternal health or inequalities in maternal health in China [21]. Such evidence from other LMICs is also limited and mixed [22–27]. Evidence from Brazil showed a significant association between the family physician strategy and maternal mortality [22], whilst evidence from Iran found no such association [23]. Studies from China have evaluated the impacts of single PHC policies on health service utilisation, such as family physicians [28, 29] and gatekeeping [30, 31], and were mostly from megacities [28, 30]. Few studies are from less-developed regions or evaluate the policy impacts on maternal health inequalities. Although there has been consistent evidence from LMICs, including China, showing the protective impacts of maternal care on maternal deaths [6, 25, 32], its impacts on maternal health inequalities are understudied. Similarly, the impacts of other primary care services, such as family physician services, on maternal health are rarely studied, and the evidence is mixed [22, 23]. To address these evidence gaps, this study uses robust quasi-experimental methods and provincial panel data to investigate the differential association between China’s PHC reforms, PHC utilisation and maternal health across regions.

## Methods

### Study design

This study is an observational longitudinal ecological study for the years 2010–2019 with province-year as the unit of analysis. Two analyses were carried out. The first is a difference-in-differences (DiD) method proposed by Callaway and Sant’Anna [35] (CS-DiD), which was adopted to assess the reform impacts on primary care utilisation and the MMR. Compared with traditional DiD methods, the CS-DiD method relaxes the assumption of constant treatment effects over time and is robust to biases raised from time-varying treatment effects of a staggered treatment [35]. Given that the PHC reforms came into effect at different times across provinces, the CS-DiD method is preferred. The CS-DiD method is also robust against some model misspecification [35]. In the second analysis, the association between primary care utilisation and the MMR was examined using a panel regression with two-way fixed effects (TWFE). The TWFE panel regression has the advantage of adjusting for unobservable and time-invariant confounders by adding year and unit fixed effects [36]. All the analyses were stratified by the HDI levels to examine the differential reform impacts and the association between primary care utilisation and the MMR across regions. Several sensitive analyses were performed.

### Data sources

This study employed routinely collected data from two annual government yearbooks, the China Health Statistic Yearbooks [10] and the China Statistic Yearbooks [37]. Data in both series of yearbooks are presented at the national and provincial levels and cover 31 provinces in mainland China. The China Health Statistic Yearbooks include information on the supply, utilisation, and expenditures of China’s health system and population health. The China Statistic Yearbooks include social and economic development data, including demography, employment and income, government financing, environment, agriculture, industry, infrastructure, trades, etc. This study extracted provincial-level data on primary care usage and maternal mortality from the China Health Statistic Yearbook. Provincial data on populations, infrastructure, and financing were obtained from the China Statistic Yearbooks. Given that data on PHC utilisation are only available since 2010, this study extracted the data between 2010 and 2019, before the onset of the Covid-19 pandemic which significantly affected healthcare use [38, 39]. This study also used the provincial Human Development Index (HDI) between 2010 and 2019 published by the United Nations Development Programme [40]. 

For exposure, we web-scrapped 31 provincial governmental websites and obtained the corresponding policy documents for the PHC reform. The policy databases were accessed from the governmental websites and searched using the term “*fen ji zhen liao*” (分级诊疗) in Mandarin (“hierarchical medical system”). The effective date of the policy was extracted using an automated web-crawler script, indicating the implementation date of the reforms in the province. One author (CC) verified the extracted implementation date with implementation date noted on the policy documents. The web-scrapping was performed using Python 3.7.

### Measurements

#### Outcomes

Maternal mortality ratio measured by the number of maternal deaths per 100,000 live births is the main outcome for both analyses.

The CS-DiD analysis also examined the reform impacts on primary care utilisation measured by number of family physician services delivered per 10,000 people, and the ratio of pregnant women who received at least one prenatal examination to the number of live births during the year.

#### Exposure

The exposure variable of the CS-DiD analysis is the implementation of the PHC reform. The reform implementation years were extracted from the 31 provincial policy documents and used as the start of each province’s PHC reform. A binary variable was constructed for each province in each calendar year as a proxy for the reform implementation. The binary takes the value of one if the province implemented the reform in/before that year (i.e., post-reform periods), or zero otherwise (i.e., pre-reform periods). All 31 provinces had implemented the reform by the end of the study period.

The exposure variables of the TWFE panel regression analysis are the utilisation of family physician services and prenatal care, measured by number of family physician services per 100 people and the percentage of of pregnant women who received at least one prenatal examination to the number of live births during the year, respectively.

#### Covariates

The CS-DiD analysis adjusted for covariates that could be associated with the PHC reform implementation or the outcomes but not affected by the reform– specifically, health insurance coverage, hospital resource allocation, transportation, and sanitation [6, 41, 42]. Therefore, the CS-DiD model adjusted for on population size (10,000 people), the percentage of people covered by national basic health insurances, number of hospitals per 10,000 people, percentage of people with access to tap water, and areas of city road per capita (square meters).

The second analysis using TWFE panel regression also adjusted for the five covariates above. Additionally, the TWFE model controlled for other types of medical service use and the availability of PHC resource using visits to PHC facilities per capita and number of PHC workforce per capita respectively to further isolate the association between prenatal care/family physician service use and the MMR.

### Statistical analysis

#### A difference-in-differences model

The first analysis employed the CS-DiD estimator [35] to estimate the impacts of the PHC reform on primary care utilisation and the MMR. This analysis used provinces in years when the reform had not-yet started as control groups and the year prior to the implementation year as the reference period. For each outcome, the reform impacts were estimated using:

$$\begin{gathered}\:{Y_{it}} = \widetilde \alpha _1^{g,t} + \widetilde \alpha _2^{g,t}{G_g} + \widetilde \alpha _3^{g,t} \cdot \:1\left\{ {T = t} \right\} + \hfill \\\,\,\,\,\,\,\,\,\,\,{\widetilde \beta ^{g,t}} \cdot \:\left( {{G_g} \times \:1\left\{ {T = t} \right\}} \right) + \widetilde \gamma \cdot \:X + {\widetilde \varepsilon ^{g,t}} \hfill \\ \end{gathered} $$

where $\:{Y}_{it}$ is the outcome of interest (i.e., primary care utilisation and the MMR) for province $\:i$ in calendar year $\:t$. $\:{G}_{g}$ is a set of binary variables which indicate whether a province first started the reform in year $\:g$. $\:T=t$ indicates the calendar year. $\:X$ is a set of pre-treatment covariates condition on which the parallel trend assumption holds. $\:{\stackrel{\sim}{\beta\:}}^{g,t}$ is the coefficient of interest, interpreted as the average reform effects for provinces with a reform implementation year $\:g$ in calendar year$\:t$. ${\widetilde \varepsilon ^{g,t}}$ is the stochastic residual term. The standard error was clustered at the provincial level. For identification, the outcome regression method was used in the CS-DiD model, which uses pre-treatment covariates of the control groups to model the expectation of the outcome changes conditional on the covariates. Since all groups were treated at the end of the observed periods, the CS-DiD automatically dropped the last treated groups from the estimation.

#### A panel regression model with two-way fixed effects

The second analysis is a TWFE panel regression model to investigate the association between primary care utilisation and the MMR:

$$\:{Y_{it}} = \alpha \: + {\beta _0}{X_{it}} + {\beta _1}{Z_{it}} + {\lambda _i} + {\delta _t} + {\varepsilon _{it}}$$

where $\:{Y}_{it}$ is the MMR in province $\:i$ at year $\:t$. $\:{\varvec{X}}_{\varvec{i}t}$ represents a set of time-varying covariates at the province level. $\:{\varvec{Z}}_{it}$ represents the main variables of interests, namely, the utilisation of family physician services and prenatal care. The set of parameters of interest are $\:{\varvec{\beta\:}}_{1}$ which captures the association between the utilisation and the MMR. $\:{\lambda\:}_{i}$ and $\:{\delta\:}_{t}$ are province and time fixed effects to control for time-invariant unobservable province-level confounders and time-specific effects respectively. The residual term $\:{\epsilon\:}_{it}$ was the stochastic disturbance term capturing any remaining unobserved confounders and assumed normally distributed and mean zero. The robust standard error was clustered at the province level.      

All analyses were stratified by the HDI level of the provinces to explore the differential association between the PHC reform, primary care utilisation, and the MMR across regions. HDI is a comprehensive indicator that measures the education, income level, and life expectancy of a region [40] and often associated with the MMR [43]. The pre-reform median HDI was calculated using the provincial HDI in 2010. Provinces whose HDI in 2010 was below the median HDI were categorised as low-HDI provinces, or as high-HDI provinces otherwise. A z-statistic test was conducted to test whether the estimated coefficients of the reform impacts among the two groups have statistical significance [44]. 

#### Robustness tests

The validity of CS-DiD estimation depends on the assumption of conditional parallel trend, which can be tested using an “event-study-type estimand” [35]. We used this estimand to test the conditional parallel trend assumption. If the assumption holds, the coefficients of the estimands during the pre-reform periods should be close to zero and statistically non-significant [35]. To further check the robustness of the CS-DiD analyses, the analyses were repeated using a control outcome, traffic-related deaths per 10,000 people. Control outcomes are anticipated to have no treatment effect and used to assess residual biases and improve internal validity of causal inference [45]. No reform impact on traffic-related deaths was expected. This study performed several tests on the appropriateness of our model selection. We applied random effects and pooled effects to the panel regression models. The two alternative models were then compared with the TWFE model using Hausman test [46]. A stepwise TWFE model was performed to examine the existence of collinearity and omitted variables. As a sensitive analysis for the HDI subgroup, the analyses were repeated using administrative division (i.e., eastern, central, and western China).

All analyses were conducted in R version 4.2.1. The CS-DiD analyses were conducted using *did* package, and all panel regression models using *plm* package.

## Results

A total of 310 observations from 31 provinces between 2010 and 2019 were included in the analysis. The provincial mean annual MMR was 21.23 per 100,000 live births during 2010–2014 which declined to 15.18 during 2015–2020 (Table 1). Over the same periods, the mean MMR among low-HDI provinces declined faster by 9.76 per 100,000 live births than in high-HDI provinces (by 2.56 per 100,000 live births). Regarding primary care utilisation, the use of family physician services and prenatal care increased during 2010 and 2019. Low-HDI provinces saw a larger increase in the use of family physician services and prenatal care than high-HDI provinces. Both groups achieved over 95% coverage of prenatal care by 2019. Meanwhile, the use of family physician services remained low, with a large standard deviation indicating drastic variations across provinces.

**Table 1:** Summary statistics of the 31 provinces before and after china’s primary health care reforms grouped by human development index

	2010–2014				2015–2019			
	Low-HDI provinces (*N* = 75)		High-HDI provinces (*N* = 80)		Low-HDI provinces (*N* = 75)		High-HDI provinces (*N* = 80)	
	Mean	SD	Mean	SD	Mean	SD	Mean	SD
Maternal mortality ratio (per 100,000 live births)	31.03	36.43	12.03	6.28	21.27	19.33	9.47	4.15
Number of family physician services delivered per 10,000 people	157.31	108.54	215.67	139.43	342.19	442.63	258.09	133.97
Prenatal services (%)	92.57	7.95	95.89	3.59	95.57	3.91	97.53	1.08
Visits to PHC facilities per capita	2.62	0.65	2.88	0.74	2.71	0.68	3.14	0.96
Number of PHC workforce per 10,000 people	25.73	6.01	24.72	4.15	28.49	7.47	27.60	4.01
Number of hospitals per 10,000 people	0.20	0.08	0.19	0.06	0.26	0.09	0.24	0.07
People covered by basic health insurance (%)	0.29	0.14	0.50	0.20	0.69	0.34	0.77	0.24
Human Development Index	0.66	0.05	0.74	0.04	0.70	0.04	0.78	0.04
Population size (10,000 people)	4329.36	2718.33	4365.95	2783.43	4455.48	2778.61	4497.29	2893.03
Percentage of people with access to tap water (%)	95.31	3.82	97.78	2.74	96.75	4.18	98.70	1.63
Areas of city road per capita (square meters)	13.87	3.12	14.46	5.17	16.95	3.66	16.04	5.51

Notes: PHC for primary health care. HDI for Human Development Index. SD for standard deviation

### The PHC reform impacts on PHC utilisation and the MMR

Columns (1) and (2) in Table 2 show the estimated associations between the PHC reform and PHC utilisation and MMR among high-HDI provinces and low-HDI provinces respectively. The PHC reform was significantly associated with a reduction in maternal deaths by 9.64 per 100,000 live births per year (95% CI 0.28–19.01) in low-HDI provinces. The reform also had a significant association with the increasing utilisation of family physician services (59.66 per 10,000 people per year, 95% CI 32.82–86.51) and prenatal care (3.17% points per year, 95% CI 1.78–4.55) in low-HDI provinces. In contrast, the reform had no significant impact on the utilisation of family physician services, prenatal care, or the MMR in high-HDI provinces. The results from the z-statistic suggest that there was a significant difference between the estimated reform impacts in low- and high-HDI provinces (Column (3) in Table 2).

**Table 2:** The CS-DiD estimations of china’s primary health care reform impacts of health service utilisation and the MMRs by low- and high-HDI provinces

Outcomes	High-HDI	Low-HDI	*p* value
	(1)	(2)	(3)
Maternal mortality ratios (per 100,000 live births)	2.07	-9.64*	0.02
	[-0.19; 4.32]	[-19.01; -0.28]	
Family physician services delivered (per 10,000 people)	-13.39	59.66*	< 0.001
	[-49.92; 23.13]	[32.82; 86.51]	
Prenatal services (%)	-0.34	3.17*	< 0.001
	[-0.78; 0.11]	[1.78; 4.55]	
**Control outcomes**			
Traffic-related deaths (per 10,000 people)	-0.10	-0.06	
	[-0.56; 0.36]	[-0.18; 0.06]	

Notes: * *p* < 0.05, ** *p* < 0.01, *** *p* < 0.001. p-value is for the significance of the z statistics of the two coefficients for high-HDI and low-HDI provinces, and the null hypothesis is that the two coefficients have no significant difference. PHC for primary health care. All models were adjusted for population size (10,000 people), areas of city road per capita, percentage of people with access to tap water, number of hospitals per 10,000 people, and percentage of people covered by basic health insurance. Standard errors were clustered at provincial level. Outcome regression was used as the estimation method to relax parallel trends assumption. Point estimates and simultaneous 95% confidence intervals were reported

### The association between PHC utilisation and the MMR

**Fig. 2:**
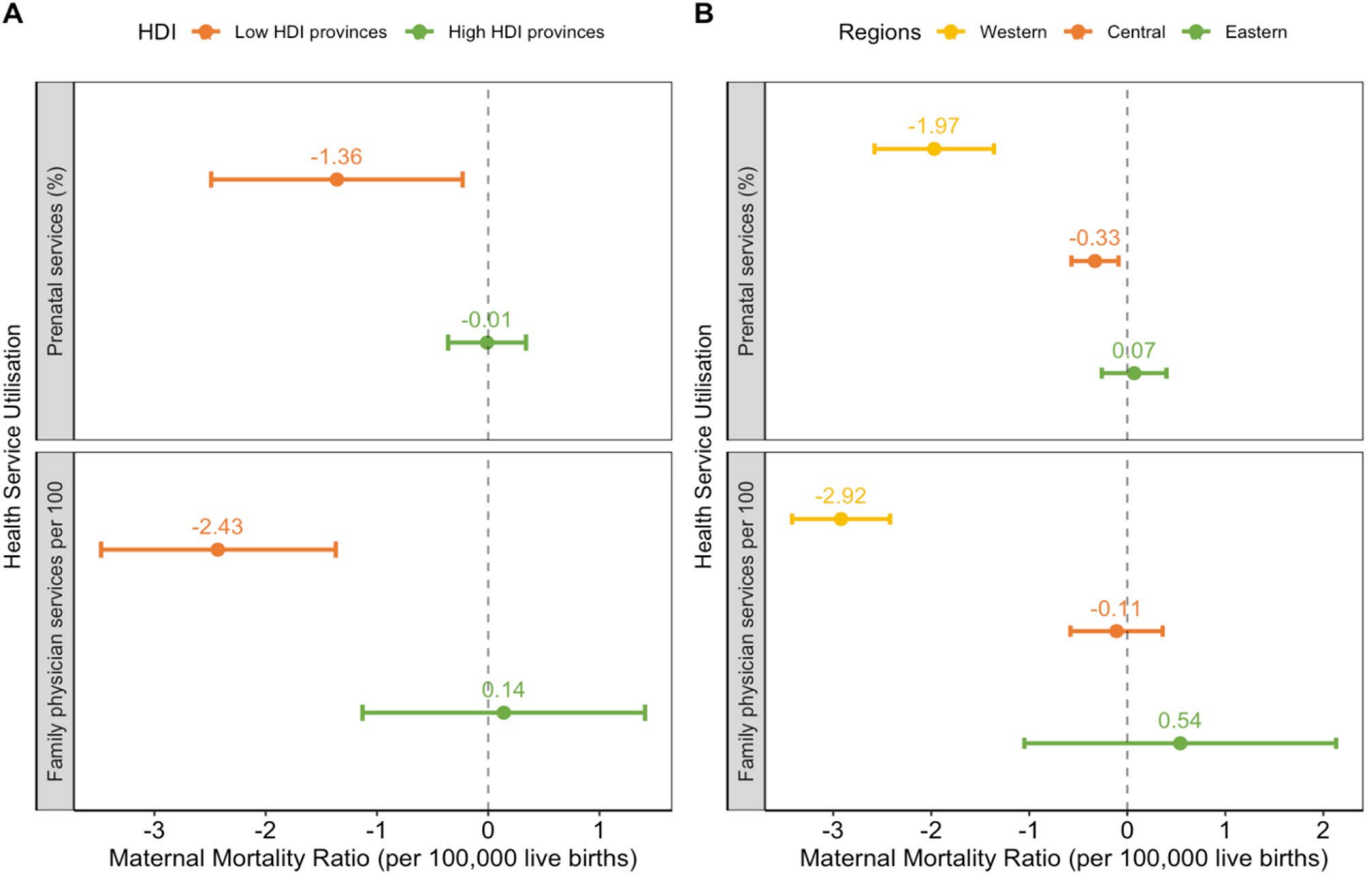
The panel regression estimations of the association between the use of prenatal care and family physician services and the maternal mortality ratio across different regions in China, 2010–2019. Notes: HDI for Human Development Index. The association were estimated using panel regression with two-way fixed effects. All models were adjusted for number of visits to PHC facilities, population size (10,000 people), areas of city road per capita, percentage of people with access to tap water, number of hospitals per 10,000 people, number of PHC workforce per 10,000 people, and percentage of people covered by basic health insurance. Standard errors were clustered at the provincial level. 95% confidence intervals were reported

Panel A in Fig. 2 compares the differential association between the utilisation of family physician services and prenatal care and the MMR between the low- and high-HDI provinces. In low-HDI provinces, utilisation of prenatal care and family physician services had a significant negative association with the MMR. A 1% point increase in prenatal care per year was associated with a reduction of 1.36 (95% CI 0.23–2.49) maternal deaths per 100,000 live births per year. An increase of one family physician visit per 100 people per year was associated with a reduction of 2.43 (95% CI 1.37–3.48) maternal deaths per 100,000 live births per year. In contrast, no association was found between the MMR and either the use of prenatal care or family physician services in high-HDI provinces.

A similar pattern was found across eastern, central and western China, with the largest reduction in the MMR found in western China (Panel B in Fig. 2). A 1% point increase in prenatal care utilisation was significantly associated with a reduction of 1.97 (95% CI 1.36–2.58) maternal deaths per 100,000 live deaths per year in western China. A smaller but significant association was found in central China (0.33 maternal deaths per 100,000 live births per year, 95% CI 0.09–0.57), whist this association was not found in eastern China. An increase of one family physician visit per 100 people per year was associated with a reduction of 2.92 (95% CI 2.42–3.42) maternal deaths per 100,000 live births per year in western China. However, no association between family physician visits and the MMR was found in central or eastern China.

### Robustness tests

Figure S1 in the appendix shows that all coefficients for the per-reform periods were not statistically significant, indicating that the conditional parallel trend assumption holds. Table 2 shows the estimated reform impacts on the control outcome (i.e., traffic-related deaths) using the CS-DiD method. There was no significant reform impact on traffic-related deaths. Table S1 in the appendix shows the estimated association between the primary care utilisation and the MMR in low- and high-HDI provinces using the fixed effects, random effects, and pooled effects models. The three models show a consistent direction of the association between primary care utilisation and the MMR. No association between visits to PHC facilities and the MMR were found in any of the models. The results from the Hausman test rejected the null hypothesis, indicating that the TWFE models were more efficient than the two alternative models. Table S2 in the appendix shows the results of the stepwise TWFE models. The direction and magnitude of the association between the use of family physician services and prenatal care and the MMR were consistent across the models, indicating no presence of collinearity and omitted variables.

## Discussion

China’s PHC reforms were associated with increases in the utilisation of prenatal care and family physician services and decreases in MMR in low-HDI provinces. These two services were also associated with the MMR after controlling for changes in the health resource inputs, suggests a likely pathway of the PHC reforms. In contrast, no reform impact was found in high-HDI provinces, nor was primary care utilisation associated with the MMR in those provinces. The distinct findings between the low- and high-HDI provinces suggest that China’s PHC reform and primary care utilisation narrowed inequalities in maternal health between regions.

It is not surprising that this study found concentrated reform impacts on increases in the utilisation of family physician services and prenatal care in less-developed areas, given that the reform goal was to deliver more equitable primary care services. The pro-equity impacts of the PHC reform on primary care utilisation have been confirmed in other population groups (e.g., older adults or people with chronic diseases) [20, 21], but not among pregnant women. Pregnant women from less-developed provinces often had more geographical and financial barriers to maternal health services [47, 48], leading to lower maternal care use [49, 50] and a higher MMR [6, 48] than those in developed provinces. These inequalities were targeted in the reform. For instance, the numbers of family physicians had higher growth rates in central and western China than eastern China [51]. Family physicians shared responsibility of delivering free essential maternal care packages with traditional midwives and PHC nursesf. They also provide home visits, consultations, prescriptions, and referrals for pregnant women. These community-level primary care services had higher reimbursement rates than hospitals and required little travel, making primary care more affordable and accessible to pregnant women in less-developed regions.

This study shows that the PHC reform was associated with a lower MMR in less-developed areas. Impacts of PHC reforms on the MMR are understudied in China, and such evidence from other LMICs is mixed [22–27]. Our finding is consistent with the observed greatest decline in the MMR in resource-constrained regions in China [52], contributing evidence on the pro-equity reform impacts on maternal health. This study also shows that the increased use of family physician services and prenatal care were associated with the reduction in the MMR in low-HDI provinces. This finding aligns with existing evidence on the protective impacts of maternal care use on maternal health [25, 53, 54], and underlines that this impact disproportionately benefits resource-constrained regions. Whilst existing evidence on the association between family physician utilisation and the MMR is limited and mixed [22, 23], the positive association found in this study is plausible. Utilisation of prenatal care and family physician services allows pregnant women to identify risk factors [11, 12, 14], access timely treatments [13, 14] and facility births [12]and thereby have safe births [6]. The disproportional increases in prenatal care use in less-developed provinces found in this study could enable these positive changes and address the unmet health needs of pregnant women in these provinces [55]. Additionally, the integrated two-way referral system introduced in the reform empowered PHC facilities in less-developed areas with personnel and technical supports from hospitals in developed regions [19], which also plausibly explains the findings.

The PHC reform or primary care utilisation was not associated with changes in MMR in high-HDI provinces or eastern China. These areas often have better access to hospital care and a lower MMR than low-HDI provinces or central/western China. The median MMR of the high-HDI provinces was 9.5 in 2014 (i.e., before the PHC reform) [10]a similar level to high-income countries like the United Kingdom [56]. In the same year, the average prenatal care utilisation rates in eastern China reached 97.24%.^10^ The low maternal mortality and high coverage of maternal care left limited improvement space for these developed regions. At the current level of utilisation of maternal care, further maternal health gains in these low-MMR regions require high-quality maternity care [57–59]. This need is in contrast with challenges faced by primary care providers in China, such as poor performance [60], limited health services and medicines [61, 62] and a lack of skilled workforce [63–66]. Without care quality improvements, maternal health improvement in the developed regions can be stalled.

This study offers important policy implications. Like China, many LMICs regard primary care providers as the main providers of maternal health services [67]. China’s reform highlights effective PHC strategies for improving maternal health in under-resourced regions and reducing maternal health inequalities across regions. First, convenient accessibility and minimal individual payments can incentivise the use of maternal care provide by PHC providers. Second, an integrated two-way referral system can facilitate collaboration between PHC providers and higher-level hospitals, increase facility births and maternal care continuity, and reduce preventable maternal deaths. On the other hand, the limited impact among the developed regions found in this study underscores challenges in attaining maternal health gains at a low-level MMR. As China gradually enters a low maternal mortality era, the lack of quality improvements in maternal care may hinder further maternal health improvements. One study warns that China’s maternal health improvement has slowed down in the past few decades, and the low maternal mortality in developed areas have posed new challenges to the health system [57]. Such plateau status was also witnessed in other low-MMR countries/regions [68]. Whilst it is crucial to expand the coverage of PHC-oriented community-level maternity care, policy makers in China and other LMICs should also be prepared for new challenges in a low-MMR era and put more efforts in care quality improvements.

This study has several strengths. Prior to this study, there was an important evidence gap in how China’s PHC reforms in recent years have affected maternal health and its inequality across regions. Our study addresses these gaps using routinely collected health administrative data and highlights the differential reform impacts on maternal health across different regions. This study triangulates the protective impacts of primary care utilisation against maternal health inequalities. This study used web-scrapping to identify the reform implementation at the provincial level, more accurate than previous studies. Our study design exploited the staggered implementation and used advanced statistical models that are robust to time-varying treatment effects and unobservable time-invariant confounders. The use of control outcomes in this study also increases the internal validity of our findings.

This study is subject to limitations. The analysis in this study is restrained by the scope and availability of yearbook data. The Chinese Yearbook data only included provincial-level data, and prefecture-level data were not available, preventing analyses with higher geographical resolutions. Better and more sensitive outcome measurements would also be beneficial for a more in-depth interpretation of the findings. For example, the yearbooks do not include any data on effective coverage of maternal health services, such as 4 + times prenatal visits. The number of visits to PHC facilities cannot distinguish the different purposes for visiting PHC facilities (e.g., whether to seek for treatments, essential medicines, or referral letters). Due to the nationwide roll-out of the reform, not-treated groups are not available for analysis. Instead, we used not-yet-treated groups as control groups in our analysis, but this posed a threat of collinearity. To address this issue, the data from the last-enrolled provinces at the last time points were automatically excluded from the CS-DID analyses. A probable consequence is an underestimation of the reform impacts. Lastly, this study is prone to ecological fallacy [69]. The provincial-level aggregated data used in this study cannot reflect individual’s exposure to the reform or their health service utilisation. The findings from this study should only be interpreted at the provincial level and not be used to infer individual-level reform impacts.

## Conclusion

China’s PHC reform and the utilisation of family physician services and prenatal care were associated with improved maternal health in lower developed regions. However, this association was not found in well-resourced regions. Policymakers in China should continue PHC strengthening in resource-restrained areas to further reduce maternal health inequalities while facilitate system and quality improvement for maternal health gains in developed areas.

## Electronic supplementary material

Below is the link to the electronic supplementary material.

Supplementary Material 1

## Data Availability

The datasets supporting the conclusions of this article are available in the National Bureau of Statistics of China Statistical Database repository, https://www.stats.gov.cn/english/Statisticaldata/yearbook/. Non-academic users can access the data by submitting data requests.

## Declarations

### Patient and public involvement

Patients or the public were not involved in the design, or conduct, or reporting, or dissemination plans of our research.
