# Supplementary material for: The association between china’s primary health care reform and inequalities in primary care utilisation and maternal mortality: a quasi-experimental longitudinal study from 2010 to 2019
Source: Int J Equity Health. 2025 Jun 13;24:174. doi: 10.1186/s12939-025-02541-z (PMC12164202; doi:10.1186/s12939-025-02541-z)
Supplement: Supplementary file 1 — Supplementary Material 1 [file 12939_2025_2541_MOESM1_ESM.docx]

**Appendixes for article: *The association between China’s primary health care reform and inequalities in primary care utilisation and maternal mortality: a quasi-experimental longitudinal study from 2010 to 2019***


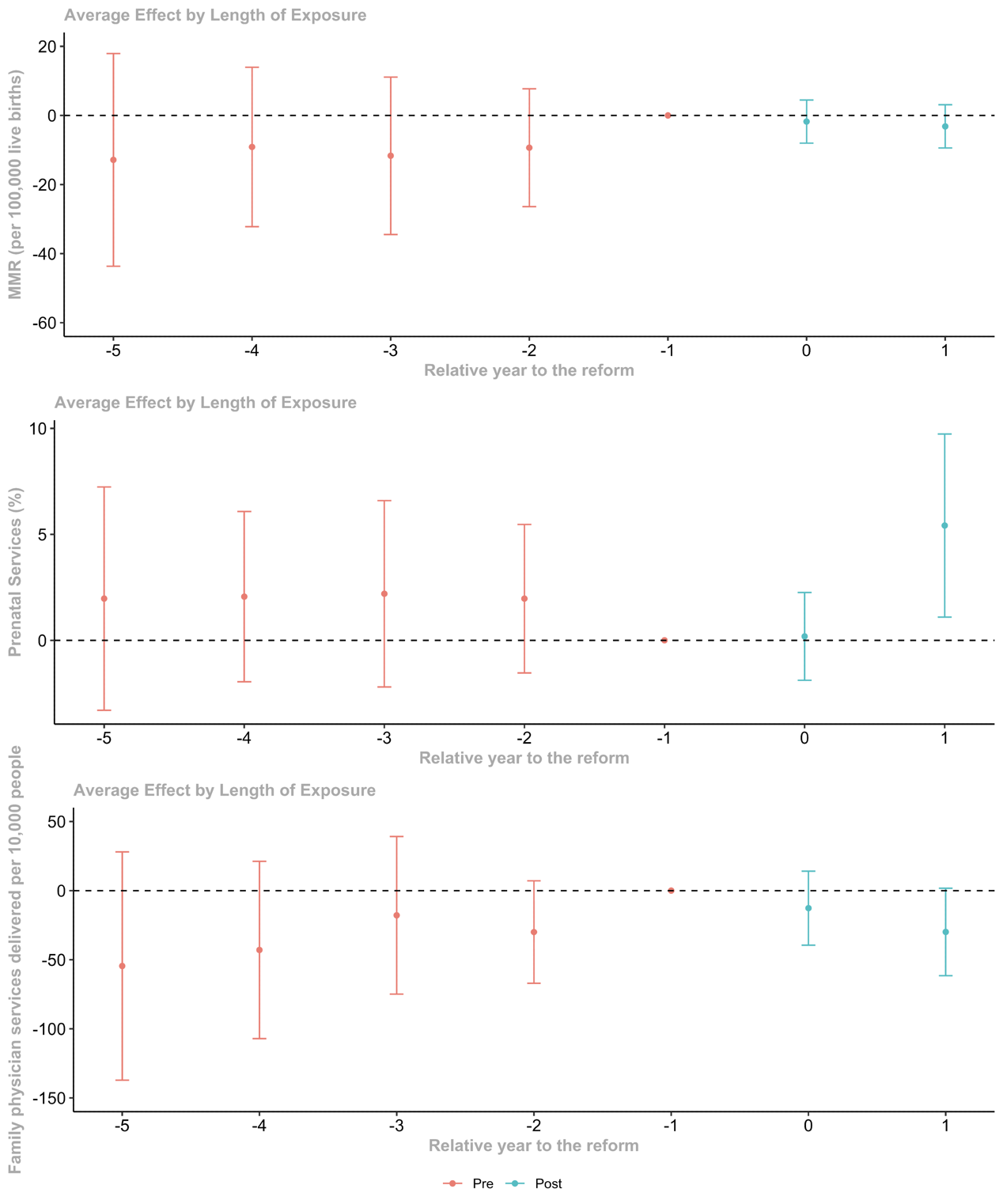


Figure S1 Event-study-type estimations using CS-DiD to test the conditional parallel trend assumption

Notes: MMR for the maternal mortality ratio. CS-DiD for the difference-in-differences method proposed by Callaway and Sant’Anna. Pre for pre-reform periods, and post for post-reform. All models were adjusted for population size (10,000 people), areas of city road per capita, percentage of people with access to tap water, number of hospitals per 10,000 people, and percentage of people covered by basic health insurance. Standard errors were clustered at the provincial level. Outcome regression was used as the estimation method to relax parallel trends assumption. Point estimates and simultaneous 95% confidence intervals were reported.

|  | | | | | **Maternal mortality ratio (per 100,000 live births)** | | | | |
| --- | --- | --- | --- | --- | --- | --- | --- | --- | --- |
|  | | | Low HDI provinces | | | | High HDI provinces | | |
|  | Fixed effects model | Random effects model | | Pooled model | | Fixed effects model | | Random effects model | Pooled model |
| Intercept |  | 267.35*** | | 325.91*** | |  | | 138.73*** | 142.17*** |
|  |  | [204.12, 330.58] | | [255.08, 396.75] | |  | | [104.01, 173.45] | [111.24, 173.09] |
| Prenatal Services (%) | -1.36* | -2.00*** | | -2.55*** | | -0.01 | | -0.1 | -0.23* |
|  | [-2.49; -0.23] | [-2.39, -1.62] | | [-2.93, -2.16] | | [-0.36; 0.34] | | [-0.31, 0.11] | [-0.43, -0.03] |
| Family physician services delivered per 100 people | -2.43*** | -2.25*** | | -2.38*** | | 0.14 | | -0.07 | -0.36 |
|  | [-3.48; -1.37] | [-2.90, -1.60] | | [-3.20, -1.56] | | [-1.13; 1.41] | | [-0.61, 0.48] | [-0.83, 0.11] |
| Visits to PHC facilities per capita | 2.38 | 1.06 | | -0.83 | | 3.05 | | -1.57* | -1.97*** |
|  | [-8.56; 13.33] | [-6.13, 8.24] | | [-5.27, 3.61] | | [-0.21; 6.32] | | [-2.87, -0.26] | [-2.95, -1.00] |
| PHC workforce per 10,000 people | -1.45* | -0.41 | | 1.18*** | | -0.48 | | 0.09 | 0.20* |
|  | [-2.54; -0.37] | [-1.03, 0.22] | | [0.70, 1.67] | | [-1.34; 0.37] | | [-0.15, 0.32] | [0.04, 0.36] |
| Areas of city roads per capita | 0.06 | 0.16 | | 0.03 | | 0.21 | | 0.17 | 0.15* |
|  | [-0.85; 0.97] | [-0.62, 0.95] | | [-0.59, 0.65] | | [-0.27; 0.70] | | [-0.02, 0.37] | [0.03, 0.27] |
| People with access to water (%) | 0.002 | -0.29 | | -0.84** | | -0.79 | | -1.16*** | -1.07*** |
|  | [-0.32; 0.31] | [-0.79, 0.22] | | [-1.43, -0.25] | | [-1.63; 0.04] | | [-1.50, -0.81] | [-1.37, -0.77] |
| Hospitals per 1,000 people | -0.7 | 2.71 | | 5.67*** | | -0.87 | | -0.72 | -0.93 |
|  | [-9.74; 8.33] | [-2.32, 7.73] | | [2.48, 8.87] | | [-4.91; 3.18] | | [-2.44, 1.01] | [-2.16, 0.29] |
| People covered by basic health insurances (%) | -4.66 | 0.92 | | -4.7 | | -0.35 | | -1.97 | -0.62 |
|  | [-10.89; 1.58] | [-5.73, 7.57] | | [-12.66, 3.27] | | [-3.47; 2.77] | | [-4.51, 0.57] | [-2.94, 1.69] |
| Population size | 3.61 | -0.48*** | | -0.33*** | | -0.17 | | -0.05 | -0.04** |
|  | [-1.30; 8.52] | [-0.72, -0.23] | | [-0.44, -0.23] | | [-0.97; 0.63] | | [-0.09, 0.00] | [-0.07, -0.02] |
| Observations | 150 | 150 | | 150 | | 160 | | 160 | 160 |
| R2 | 0.96626 | 0.656 | | 0.847 | | 0.79068 | | 0.426 | 0.641 |
| Within R2/Adjusted R2 | 0.74237 | 0.634 | | 0.837 | | 0.16369 | | 0.392 | 0.619 |
| AIC | 997.4 | 1077.1 | | 1180.2 | | 814.2 | | 817.1 | 854.6 |
| RMSE | 5.4 | 8.15 | | 11.49 | | 2.49 | | 2.9 | 3.26 |
| Wald | 71.3, p < 0.001 | 267, p < 0.001 | | 775, p < 0.001 | | 2.21 p = 0.02 | | 112, p < 0.001 | 268, p < 0.001 |
| Hausman Test |  | 851, p < 0.001 | | 5030, p < 0.001 | |  | | 17, p=0.05 | 165, p < 0.001 |

**Table S1 The estimated association between the utilisation of prenatal care and family physician services and the MMR using fixed effects, random effects, and pooled effects model by the HDI groups.**

Notes: * p < 0.05, ** p < 0.01, *** p < 0.001. PHC for primary health care. The fixed effects models were adjusted for year and province fixed effects. Standard errors were clustered at the provincial level for all models. 95% confidence intervals were reported.

|  | **Maternal mortality ratio (per 100,000 live births)** | | | | | | |
| --- | --- | --- | --- | --- | --- | --- | --- |
|  | Low HDI provinces | | | | | | |
| Prenatal Services (%) | -1.48. | -1.31* | -1.31* | -1.32* | -1.31* | -1.31* | -1.36* |
|  | [-3.27; 0.30] | [-2.56; -0.05] | [-2.55; -0.06] | [-2.58; -0.05] | [-2.56; -0.06] | [-2.57; -0.06] | [-2.49; -0.23] |
| Family physician services delivered per 100 people | -2.93*** | -2.53*** | -2.52** | -2.51** | -2.51*** | -2.50*** | -2.43*** |
|  | [-4.30; -1.56] | [-3.79; -1.27] | [-3.85; -1.20] | [-3.83; -1.19] | [-3.81; -1.22] | [-3.79; -1.21] | [-3.48; -1.37] |
| Visits to PHC facilities per capita | 0.84 | 5.92 | 5.92 | 5.87 | 5.17 | 5.13 | 2.38 |
|  | [-8.10; 9.77] | [-4.67; 16.51] | [-4.62; 16.45] | [-4.59; 16.33] | [-5.92; 16.26] | [-6.18; 16.44] | [-8.56; 13.33] |
| PHC workforce per 10,000 people |  | -1.72* | -1.72* | -1.74* | -1.66* | -1.67* | -1.45* |
|  |  | [-3.06; -0.39] | [-3.06; -0.39] | [-3.09; -0.39] | [-2.98; -0.34] | [-2.99; -0.34] | [-2.54; -0.37] |
| Areas of city roads per capita |  |  | 0.02 | 0.02 | 0.01 | 0 | 0.06 |
|  |  |  | [-0.72; 0.75] | [-0.72; 0.77] | [-0.79; 0.81] | [-0.80; 0.80] | [-0.85; 0.97] |
| People with access to water (%) |  |  |  | -0.06 | -0.06 | -0.07 | 0 |
|  |  |  |  | [-0.31; 0.19] | [-0.34; 0.21] | [-0.35; 0.22] | [-0.32; 0.31] |
| Hospitals per 1,000 people |  |  |  |  | -2.03 | -2.04 | -0.7 |
|  |  |  |  |  | [-12.04; 7.98] | [-12.21; 8.12] | [-9.74; 8.33] |
| People covered by basic health insurances (%) |  |  |  |  |  | -2.09 | -4.66 |
|  |  |  |  |  |  | [-5.54; 1.37] | [-10.89; 1.58] |
| Population size |  |  |  |  |  |  | 3.61 |
|  |  |  |  |  |  |  | [-1.30; 8.52] |
| Year fixed effects | Yes | Yes | Yes | Yes | Yes | Yes | Yes |
| Province fixed effects | Yes | Yes | Yes | Yes | Yes | Yes | Yes |
| Observations | 150 | 150 | 150 | 150 | 150 | 150 | 150 |
| R2 | 0.9491 | 0.9628 | 0.9628 | 0.96282 | 0.963 | 0.96309 | 0.96626 |
| Within R2 | 0.61133 | 0.71592 | 0.71593 | 0.71612 | 0.7175 | 0.71819 | 0.74237 |

| **(Continued)** | **Maternal mortality ratio (per 100,000 live births)** | | | | | | |
| --- | --- | --- | --- | --- | --- | --- | --- |
|  | High HDI provinces | | | | | | |
| Prenatal Services (%) | -0.04 | -0.02 | -0.02 | 0.02 | 0.01 | 0.01 | -0.01 |
|  | [-0.42; 0.34] | [-0.41; 0.38] | [-0.41; 0.36] | [-0.26; 0.30] | [-0.29; 0.31] | [-0.30; 0.31] | [-0.36; 0.34] |
| Family physician services delivered per 100 people | 0.05 | -0.07 | -0.06 | 0.06 | 0.06 | 0.06 | 0.14 |
|  | [-0.90; 0.99] | [-1.15; 1.00] | [-1.14; 1.01] | [-0.99; 1.10] | [-1.01; 1.12] | [-0.98; 1.11] | [-1.13; 1.41] |
| Visits to PHC facilities per capita | 2.14 | 4.56. | 4.52* | 2.87. | 2.92. | 2.86. | 3.05. |
|  | [-1.25; 5.54] | [-0.06; 9.17] | [0.10; 8.95] | [-0.52; 6.27] | [-0.46; 6.30] | [-0.42; 6.14] | [-0.21; 6.32] |
| PHC workforce per 10,000 people |  | -0.61 | -0.63 | -0.43 | -0.45 | -0.45 | -0.48 |
|  |  | [-1.51; 0.28] | [-1.60; 0.33] | [-1.33; 0.48] | [-1.38; 0.48] | [-1.36; 0.47] | [-1.34; 0.37] |
| Areas of city roads per capita |  |  | -0.09 | 0.22 | 0.22 | 0.23 | 0.21 |
|  |  |  | [-0.62; 0.44] | [-0.30; 0.74] | [-0.31; 0.75] | [-0.27; 0.74] | [-0.27; 0.70] |
| People with access to water (%) |  |  |  | -0.80* | -0.78. | -0.79. | -0.79. |
|  |  |  |  | [-1.58; -0.01] | [-1.60; 0.04] | [-1.62; 0.04] | [-1.63; 0.04] |
| Hospitals per 1,000 people |  |  |  |  | -0.62 | -0.6 | -0.87 |
|  |  |  |  |  | [-4.99; 3.75] | [-5.00; 3.80] | [-4.91; 3.18] |
| People covered by basic health insurances (%) |  |  |  |  |  | -0.34 | -0.35 |
|  |  |  |  |  |  | [-3.41; 2.73] | [-3.47; 2.77] |
| Population size |  |  |  |  |  |  | -0.17 |
|  |  |  |  |  |  |  | [-0.97; 0.63] |
| Year fixed effects | Yes | Yes | Yes | Yes | Yes | Yes | Yes |
| Province fixed effects | Yes | Yes | Yes | Yes | Yes | Yes | Yes |
| Observations | 160 | 160 | 160 | 160 | 160 | 160 | 160 |
| R2 | 0.75928 | 0.76834 | 0.76873 | 0.78993 | 0.79015 | 0.79021 | 0.79068 |
| Within R2 | 0.03822 | 0.07442 | 0.07598 | 0.16067 | 0.16156 | 0.16181 | 0.16369 |

Table S2 The stepwise panel regression estimations of the association between the utilisation of prenatal services and family physician services and the maternal mortality ratio in China, 2010-2019, grouped by HDI

Notes: * p < 0.05, ** p < 0.01, *** p < 0.001. PHC for primary health care. Standard errors were clustered at the provincial level. 95% confidence intervals were reported.
